# Supplementary material for: Coherent control of an ultrabright single spin in hexagonal boron nitride at room temperature
Source: Nat Commun. 2023 May 20;14:2893. doi: 10.1038/s41467-023-38672-6 (PMC10199889; doi:10.1038/s41467-023-38672-6)
Supplement: Supplementary file 1 — Supplementary Information [file 41467_2023_38672_MOESM1_ESM.pdf]

Supplementary Information for
Coherent control of an ultrabright single spin in hexagonal boron nitride at room temperature

Nai-Jie Guo,[1,2,3,*] Song Li,[4,*] Yuan-Ze Yang,[1,2,3,*] Xiao-Dong Zeng,[1,2,3,*] Shang Yu,[1,2,3]
Yu Meng,[1,2,3] Zhi-Peng Li,[1,2,3] Zhao-An Wang,[1,2,3] Lin-Ke Xie,[1,2,3] Rong-Chun Ge,[5]
Jun-Feng Wang,[1,2,3,5] Qiang Li,[1,2,3] Jin-Shi Xu,[1,2,3] Wei Liu,[1,2,3,†] Yi-Tao Wang,[1,2,3,‡]
Jian-Shun Tang,[1,2,3,§] Adam Gali,[4,6,¶] Chuan-Feng Li,[1,2,3,**] and Guang-Can Guo[1,2,3]

[1]*CAS Key Laboratory of Quantum Information, University of Science and Technology of China, Hefei, Anhui 230026, China.*
[2]*CAS Center For Excellence in Quantum Information and Quantum Physics, University of Science and Technology of China, Hefei, Anhui 230026, China.*
[3]*Hefei National Laboratory, University of Science and Technology of China, Hefei 230088, China*
[4]*Wigner Research Centre for Physics, H-1121 Budapest, Hungary*
[5]*College of Physics, Sichuan University, Chengdu China, 610064*
[6]*Department of Atomic Physics, Institute of Physics, Budapest University of Technology and Economics, H-1111 Budapest, Hungary*

I. SUPPLEMENTARY NOTE 1

The correction of the emission rate of Defect A. The saturated emission rate I_{sat} measured in the experiment is $\sim 3.7 \times 10^6$ counts/s. The corrected emission rate $I_{\text{sat}}^{\text{corrected}}$ can be written as $I_{\text{sat}}^{\text{corrected}} = I_{\text{sat}}/\eta$, where η is the loss coefficient. The loss coefficient η is $\sim 14.9\%$ (including the objective collection efficiency considering the reflection of the gold film (56%), fiber coupling efficiency (38%), and detection efficiency of the single photon detector (70%)). Therefore, we can calculate that $I_{\text{sat}}^{\text{corrected}} = 2.5 \times 10^7$ counts/s.

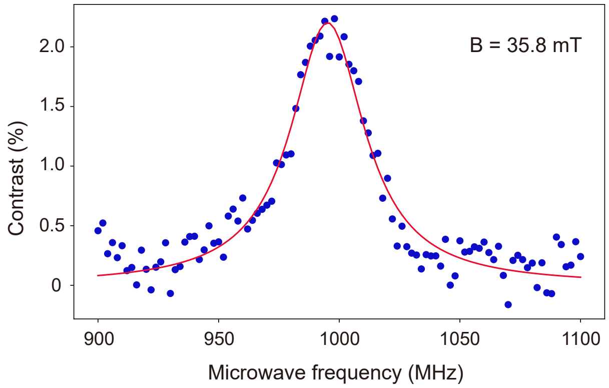

Supplementary Fig. 1: **ODMR spectrum of Defect A.** The room-temperature ODMR spectrum of Defect A at a 35.8-mT external magnetic field exhibits a positive peak at 970 MHz, whose contrast exceeds 2% and FWHM is ~ 37 MHz.

II. SUPPLEMENTARY NOTE 2

A relatively higher ODMR contrast. Supplementary Fig. 1 shows a room-temperature ODMR spectrum with a contrast of more than 2% for Defect A. This value is obtained when the sample is first tested, and the difference may be induced by the variations in experimental conditions, especially the resistance that affects the microwave radiation efficiency. Nevertheless, this higher contrast exhibits the intrinsic ability of this defect.

III. SUPPLEMENTARY NOTE 3

Magnetic-field, laser-power and microwave-power-dependent ODMR of Defect A.
Supplementary Fig. 2 shows the ODMR spectra at seven magnetic fields between 30.4∼36.1 mT at 100-μW laser power and 25-dBm microwave power, which exhibit a similar FWHM of the resonance.

Supplementary Fig. 3 shows the ODMR spectra at different laser powers and microwave powers at a 34-mT magnetic field. The FWHM of the resonance at different powers suggests the power broadening phenomenon.

The corresponding FWHM values are shown in Fig. 5 of the main text.

IV. SUPPLEMENTARY NOTE 4

Part of the measurement results of the ultrabright color centers on the array sample. Supplementary Table 1 shows nine ultrabright color centers with emission rates exceeding 0.5 MHz under 100-μW

---

*These authors contributed equally to this work.
†Electronic address: `lw691225@ustc.edu.cn`
‡Electronic address: `yitao@ustc.edu.cn`
§Electronic address: `tjs@ustc.edu.cn`
¶Electronic address: `gali.adam@wigner.hu`
**Electronic address: `cfli@ustc.edu.cn`

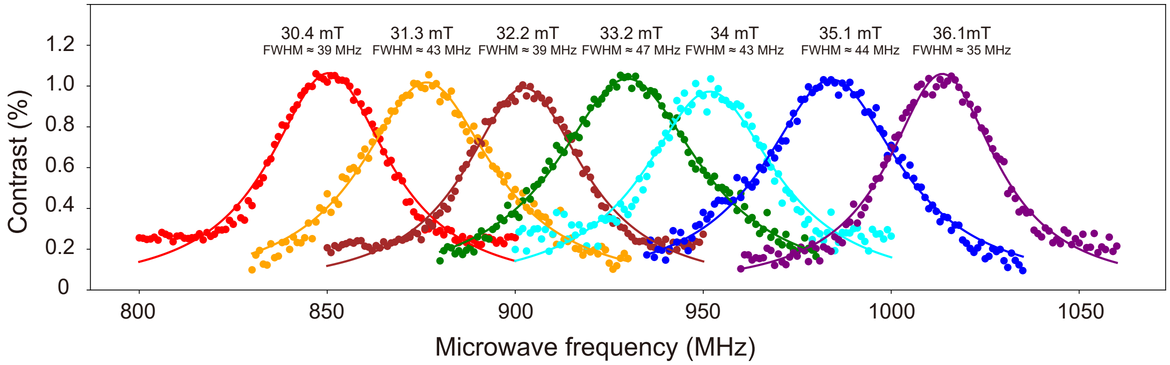

Supplementary Fig. 2: **Magnetic-field-dependent ODMR of Defect A.** We measured the ODMR spectra at seven magnetic fields between 30.4∼36.1 mT at 100-μW laser power and 25-dBm microwave power. The ODMR spectra suggest similar FWHMs of the resonance.

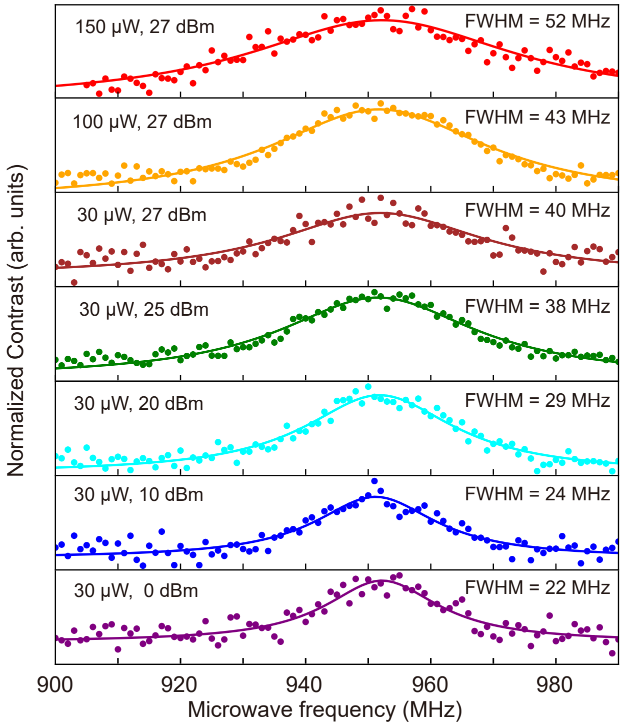

Supplementary Fig. 3: **Laser-power and microwave-power-dependent ODMR of Defect A.** We measured the ODMR spectra at different laser powers and microwave powers with a 34-mT magnetic field. The FWHM of the resonance at different powers suggests the power broadening phenomenon.

laser excitation at 532 nm, displaying a high probability of obtaining optically detectable single spins among the bright spots in the array. More importantly, these defects all exhibit consistent optical properties (ZPL at 540±10 nm) and ODMR signals.

Supplementary Table 1: List of part of the ultrabright color centers (>0.5 MHz) on the array sample under 100-μW laser excitation at 532 nm. Note: The spectra are truncated at 540 nm due to the 532-nm longpass filter, and Defect F is bleached soon after laser excitation.

label	counts (10^5 /s)	ZPL (nm)	$g^{(2)}(0)$	ODMR
Defect A	7	546	0.25	√
Defect B	16	<540	0.37	√
Defect C	12	<540	0.63	√
Defect D	20	<540	0.62	√
Defect E	6	548	0.84	×
Defect F	6	–	0.64	–
Defect G	18	540	0.8	√
Defect H	11	<540	0.62	√
Defect I	14	<540	0.74	√

V. SUPPLEMENTARY NOTE 5

A type of previously reported single spin defect. In addition to the newly found ultrabright single spin defect described here, we also find a type of single spin defect that has been reported before [1, 2], i.e., Defect J, as shown in Supplementary Fig. 4(a). The photoluminescence count of Defect J is $\sim 1 \times 10^5$ counts/s under 100-μW laser excitation, which is lower than Defect A ($\sim 7 \times 10^5$ counts/s). Supplementary Fig. 4(b) shows the room-temperature PL emission spectrum of Defect J, revealing a ZPL at $\sim$ 576 nm and PSB at $\sim$ 588 nm. The second-order autocorrelation function $g^{(2)}(\tau)$ of De-

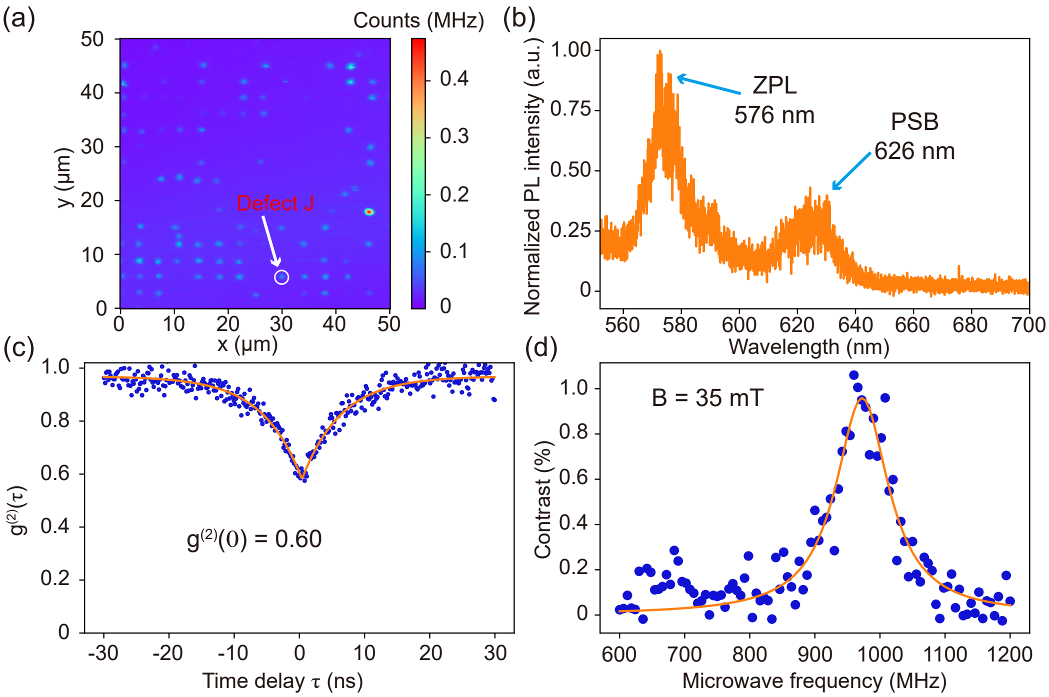

Supplementary Fig. 4: **Optical and spin properties of Defect J.** (a) 50×50-μm^2 confocal PL map of hBN arrays with 100-μW laser excitation at 532 nm. Defect J is circled in the image. (b) Room-temperature photoluminescence spectrum of Defect J under 532-nm laser excitation. ZPL is zero-phonon line and PSB is phonon sideband. (c) Second-order autocorrelation measurement $g^{(2)}(\tau)$ of Defect J which is measured at 300-μW laser power. The blue dots are the experimental data, and the orange curve is a fit yielding $g^{(2)}(0) = 0.60$. (d) Room-temperature ODMR spectrum of Defect J at 300-μW laser power and under a 35-mT external magnetic field.

fect J is presented in Supplementary Fig. 4(c), showing evidence of quantum emission but with background fluorescence contributions resulting in a $g^{(2)}(0)$ value of $\sim$ 0.60. Supplementary Fig. 4(d) displays the ODMR spectrum of Defect J under a 35-mT external magnetic field, exhibiting a positive contrast of $\sim$ 1% at 970 MHz. The optical and spin properties of Defect J are consistent with previously reported carbon-related defects [1, 2]; thus, we can classify this type of defect as a carbon-related defect. We want to note that Defect J actually does not reach the 0.5-MHz brightness threshold; therefore, it is not in our list when we calculate the probabilities in Table II of the main text.

VI. SUPPLEMENTARY NOTE 6

Other novel defects. In addition to Defect A described in the main text, we identify two different new defects that exhibit a measurable ODMR signal and are obviously distinct from Defect A, and they are denoted as Defect K and Defect L in the following. Supplementary Figs. 5(a) and (d) display the photoluminescence spectra of Defect K and Defect L at room temperature. The spectra are truncated at 540 nm due to the limitation of our 532-nm longpass filter. The ZPL of Defect K is below 540 nm. Defect L possesses a distinct ZPL at $\sim$ 546 nm and two relatively weak PSBs at $\sim$ 565 nm and $\sim$ 588 nm. We also show in Supplementary Figs. 5(b) and (e) that the second-order autocorrelation values at zero delay $g^{(2)}(0)$ for Defect K and Defect L are 0.53 and 0.39, respectively. Supplementary Figs. 5(c) and (f) show the ODMR spectra of Defect K and L at room temperature. The ODMR spectrum of Defect K exhibits six hyperfine lines and can be fitted well by a six-Lorentzian function. The results yield a hyperfine splitting constant of $\sim$ 50 MHz, which is similar to the hyperfine structure of V_B^- defects [3]. Defect L possesses a positive high contrast of 7% and a relatively wide FWHM of 116 MHz.

VII. SUPPLEMENTARY NOTE 7

The defect level diagram of the positively charged C_BO_N at C_{2v} symmetry is shown in Supplementary Fig. 6(a). There is an occupied b_2 state and empty b_2' and a_2 states in the spin majority channel (spin-up channel in the figure). The wavefunction shows an out-of-plane

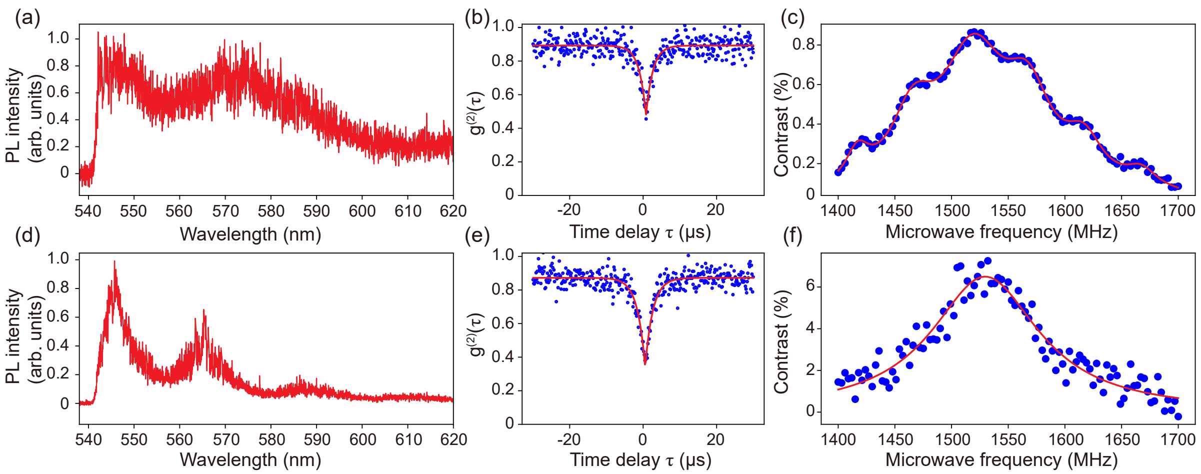

Supplementary Fig. 5: **Optical and spin properties of Defect K and Defect L.** (a) Photoluminescence spectrum of Defect K. (b) Second-order autocorrelation measurement $g^{(2)}(\tau)$ of Defect K. (c) ODMR spectrum of Defect K. (d) Photoluminescence spectrum of Defect L. (e) Second-order autocorrelation measurement $g^{(2)}(\tau)$ of Defect L. (f) ODMR spectrum of Defect L. All measurements are performed with a 532-nm longpass filter at 40-μW laser power and under a 54-mT magnetic field at room temperature.

Supplementary Table 2: Calculated hyperfine constants of the positively charged C_BO_N defect with C_{2v} symmetry in MHz. The main contribution comes from the substituted carbon, first-neighboring boron and nitrogen isotopes. The hyperfine constants are calculated for ^{11}B and ^{14}N.

atom	A_{xx}	A_{yy}	A_{zz}
^{13}C	23.94	24.12	271.48
^{11}B1	-0.94	-0.43	1.52
^{11}B2,3	-1.06	-0.55	1.78
^{11}B4	-0.44	0.13	1.36
^{11}B5,9	-2.44	0.25	2.33
^{11}B6,7	-1.55	-1.02	1.08
^{11}B8,10	-2.06	1.07	6.65
^{14}N1	-0.26	-0.23	1.05
^{14}N2	-0.50	-0.08	1.60
^{17}O	11.32	7.49	-13.53

Supplementary Table 3: Calculated hyperfine constants of the C_NC_{B3} defect in MHz. The main contribution comes from the substituted carbon, first-neighboring nitrogen and nitrogen isotopes. The hyperfine constants are calculated for ^{11}B and ^{14}N.

atom	A_{xx}	A_{yy}	A_{zz}
^{13}C	10.19	11.33	144.21
^{13}C	8.43	9.62	136.42
^{13}C	-33.54	-35.95	-56.50
^{11}B	-2.65	-2.73	-3.90
^{11}B	-0.15	1.84	6.61
^{11}B	-0.24	1.79	6.48
^{14}N	-4.58	-4.33	4.08
^{14}N	-0.23	-0.48	-2.84
^{14}N	-1.16	-5.11	-5.30
^{14}N	-4.35	-4.10	4.26
^{14}N	-0.87	-5.07	-5.24

spatial distribution, which is consistent with recent results from angular dependence analysis [4]. The transition dipole moment is oriented in plane. The hyperfine tensors are calculated for the nuclear spin active isotopes (^{13}C, ^{11}B and ^{14}N), as shown in Supplementary Table 2. Although ^{13}C has an active nuclear spin, the natural abundance is low (1.1%) and, therefore, cannot contribute to the hyperfine splitting for approximately 99% of the single spin centers (similar to oxygen with less than 0.04% abundance). Due to the C_{2v} symmetry, symmetrically equivalent atoms have the same hyperfine constants. ^{10}B (abundance 19.9%) might also occur for

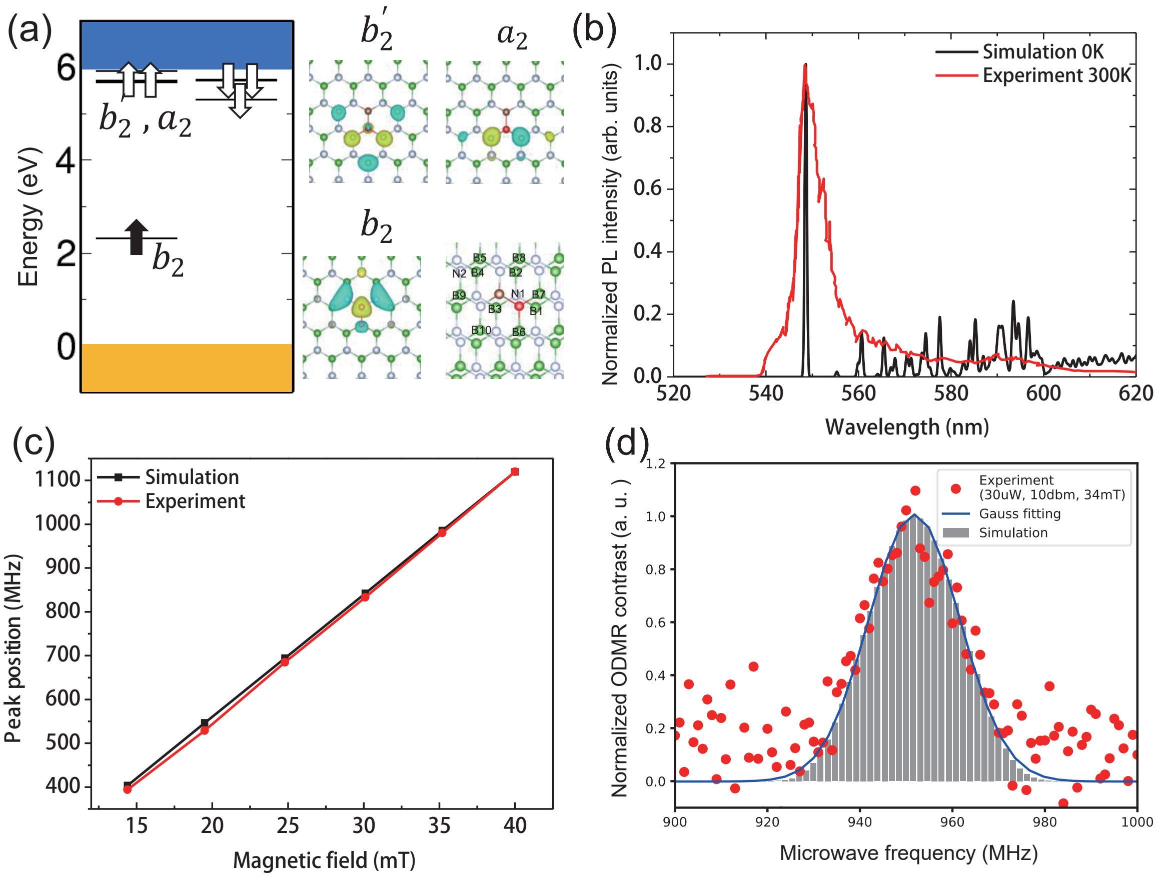

Supplementary Fig. 6: **Simulated electronic and optical properties of C_BO_N with C_{2v} symmetry.** (a) Kohn-Sham energy diagram of a positive charged C_BO_N with a localized wavefunction. The labeled atoms are the main contributors to hyperfine interactions. (b) Photoluminescence spectrum of C_BO_N at 0K. (c) Dependence of ODMR resonance frequencies on the magnetic field. (d) Comparison between the simulated and experimental ODMR signals. The model could reveal the origin of the narrow linewidth of the ODMR signal to some extent, although it is not stable and cannot match the experimental data so well.

single spin centers in the experiment. Replacing ^{11}B with ^{10}B further decreases the linewidth.

The PSB simulation is based on the Franck-Condon approximation, which can be described by the overlap between the phonon mode in ground and excited states. Here, the calculation of the luminescence spectrum requires three steps: 1. computing the electronic ground and excited state with geometry optimization by HSE functionals, 2. computing the phonon in the electronic ground state by PBE functionals, and 3. computing the overlap between the phonon modes in the electronic ground and excited states. The temperature effect on phonon is not included. The maximum of the PSB is located at approximately 580 nm. The simulated PSB is larger than the experimental result. However, the C_{2v} symmetry is unstable, and the oxygen atom makes the carbon atom move out-of-plane. This might be due to the repulsion between C_B and O_N since they both act as donors in hBN. As a consequence, the calculated ZPL energies are lower (1.71 eV) and the Debye-Waller factor is higher in the low-symmetry configuration with respect to the C_{2v} configuration, as shown in Supplementary Fig. 7, and the calculated hyperfine constants are also affected considerably in the electronic ground state.

We consider the positively charged double C_B ($2C_B$) and triple neutral C_B ($3C_B$) and find that the ZPLs are smaller than the observed ZPL [9]. The ODMR spectra are quite similar to that of the single C_B defect with a linewidth of approximately 50 MHz. Increasing the C_B-C_B distance in $2C_B$ decreases the energy difference between two defect orbitals and decreases the ZPL energy. C_2C_B has an ODMR width of approximately 27 MHz, but the ZPL is less than the observed range [7]. Recently, C_2C_N has been considered as a possible single photon emitter with a ZPL at 2.33 eV for the second optical transition [8]. The simulated ODMR linewidth is 51 MHz. This might be related to Defect L in our study with respect to power broadening. Here, we cannot exclude the possibility of C_N for the same reason. Unfortunately, this effect cannot currently be simulated. In

addition to carbon-related defects, other configurations are also calculated. For intrinsic defects, the negatively charged boron antisite (B_N) has a ZPL at approximately 2.324 eV with $S = 1.83$; however, the FWHM of the ODMR broadening is 153 MHz. The positively charged nitrogen antisite (N_B) has a ZPL energy at 2.920 eV, which is out of the ballpark of the observed ZPL energies of single photon emitters in our study.

The gyromagnetic ratios are large for both boron and nitrogen isotopes, and their interactions with the electron spin generate a broad ODMR signal. The relatively narrow ODMR linewidth in the experiment is related to the spin density localization on either nuclear spin free isotopes with high natural abundance or nuclear spin isotopes with small gyromagnetic ratio, which is followed by the spin density overlapping with neighboring boron and nitrogen atoms with nonzero nuclear spins. The likely impurity candidates are carbon, oxygen and silicon atoms. Oxygen on the boron site (O_B) has extremely high formation energy, as indicated before, and oxygen on the nitrogen site (O_N) only has one occupied state close to CBM, which cannot be responsible for the observed transition [5]. Nor is silicon substitution a good candidate since its optical excitation energy is approximately 4 eV [6], and silicon is not a common defect without intentional doping. Complex pairs are also good candidates for quantum emitters. Complex pairs with native defects, including C_BB_N, C_NN_B, C_BN_B, C_NB_N, B_NO_N, N_BO_N, largely couple to native antisite defects. Positively charged C_BO_N and C_NO_N can have a ZPL of approximately 2.3 eV depending on the distance between the pairs. The shortest-distance C_BO_N pair has an ODMR linewidth of 23 MHz in the positive charge state. We find that the positively charged C_NO_N-$\sqrt{7}a$ (a is the lattice constant of hBN) has an ODMR linewidth of 119 MHz, and the optical signal has a ZPL of 2.30 eV with $S = 2.38$ HR factor. We conclude that this defect can also be a reasonable candidate for defect L. Further detailed investigation of donor-acceptor pairs (DAPs) might reveal the nature of the single spin species in hBN, but we focus on the most characterized Defect A in our study. In Supplementary Fig. 8, we plot the singly occupied Kohn-Sham wavefunction of several defects we consider here, which is mostly associated with the spin density distribution. C_B is the defect we studied before, and the major contribution of hyperfine interactions comes from neighboring nitrogen atoms. Consequently, we speculate the narrow linewidth is due to the substitution of nitrogen with nuclear spin inactive elements discussed above. Removing one nitrogen creates the V_NC_B structure, which is a singlet. We then use hydrogen to passivate the carbon dangling bond, which is a doublet; however, hydrogen has a large hyperfine contribution. Thus, hydrogen is unlikely to exist in Defect A. The in-plane dangling bonds form an in-plane distribution wavefunction, which unavoidably interacts with the nuclear spin from boron and nitrogen, for example, in the negatively charged V_NC_B and $C_BO_NV_B$ defects. Therefore, in-plane dangling bonds should be avoided, which also means that the *sp*2 bonding type should be well preserved. Another example is C_BO_i where oxygen is an interlayer interstitial impurity. The carbon atom is pulled out of plane and transforms to the *sp*3 bonding type, which distributes the wavefunction on the nearby nitrogen atoms. The ODMR spectrum of C_NO_N-$\sqrt{7}a$ is very similar to that of C_N because the long distance between oxygen and carbon. Oxygen should be placed as the first neighbor of carbon to reduce hyperfine interactions from boron or nitride (C_BO_N). The calculated hyperfine constants are listed in Supplementary Table 2. Indeed, the hyperfine constants of the immediate two nitrogen neighbors are moderate, resulting in a relatively narrow FWHM in the ODMR spectrum. However, as indicated above, there is strong repulsion between C_B and O_N since they both act as donors in hBN. Similarly, the C_N and O_B are unlikely to form a complex, as they are both acceptors. Alternatively, carbon can be used to substitute neighboring atoms to form carbon clusters. Here, we propose the C_NC_{B3} defect, as plotted in Supplementary Fig. 9, in light of the ODMR spectra of C_2C_B. The positive charge state is a doublet and is stable when the Fermi level lies in the middle of the gap. The simulated ODMR linewidth is 28.5 MHz. However, the ZPL is 1.83 eV and $S = 1.53$. The calculated optical properties are not fully consistent with the high Debye-Waller factor and the ZPL energy at approximately 2.28 eV. Therefore, we conclude that these types of defects could be the core structures of more extended defects at which the spin density is localized, but their optical properties are perturbed by the presence of other nearby defects.

VIII. SUPPLEMENTARY NOTE 8

Structural characterization of hBN nanoparticles. Supplementary Fig. 11(a) shows the Raman spectrum of a nanoparticle with a bright spin defect on a Au substrate. The Raman spectrum exhibits a Raman shift of 1365.13 cm^{-1}, which is consistent with the previous Raman results of the hBN sample [10]. Supplementary Fig. 11(b) presents a transmission electron microscope (TEM) image of hBN nanoparticles. The TEM image exhibits an evident lattice period with d (d-spacing) equal to 0.35 nm, which is marked by the red lines and arrows in Supplementary Fig. 11(b). The theoretical hBN lattice is also displayed in the inset of Supplementary Fig. 11(b), where the theoretical d is equal to 0.37 nm along the lattice orientation indicated by the blue lines in the inset. The similar d in the TEM image and the theoretical hBN lattice indicate that the nanoparticle containing the bright spin defect should be an hBN crystal. The slight difference between the theoretical lattice and our TEM results can be attributed to the lattice strain of the hBN sample or other reasons, such as temperature.

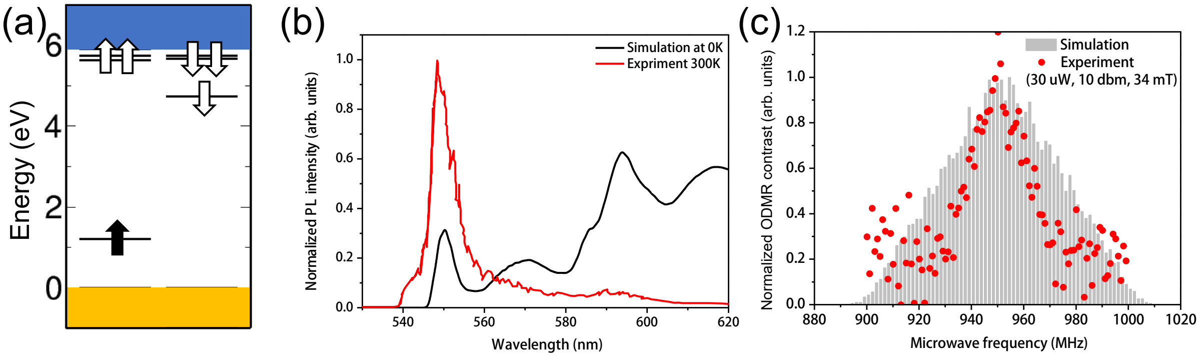

Supplementary Fig. 7: **Simulated electronic and optical properties of C_BO_N with C_{1h} symmetry.** (a) Kohn-Sham energy diagram of positive charged C_BO_N. (b) Photoluminescence spectrum of C_BO_N at 0K. (c) Comparison between the simulated and experimental ODMR signals.

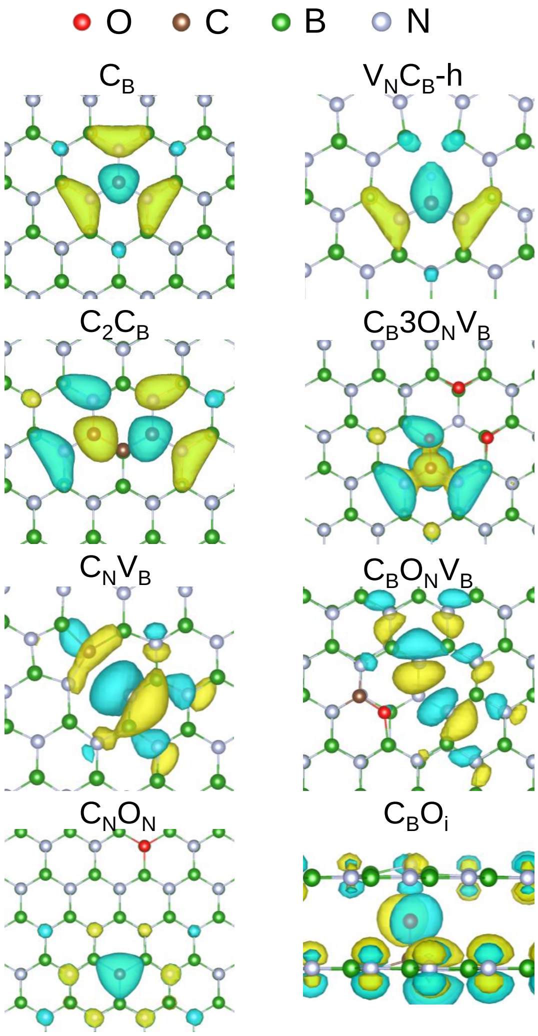

Supplementary Fig. 8: **Single spin wavefunction of proposed defects.** Pure carbon-related defects can be found in Ref. [7–9]

IX. SUPPLEMENTARY NOTE 9

Brightness statistics of the hBN defect arrays. In this work, we choose defects brighter than 0.5 MHz under 100-μW laser excitation as the reported bright spin defects, and the percentage of the hBN nanoparticles containing these bright defects with a single spin is approximately 0.1% among all hBN nanoparticles (regardless of bright or dark). Although the percentage of this bright defect is relatively low, we find that most of these bright defects (up to 85%) are isolated single spin defects with evident ODMR signals, and in addition, these bright spin defects can be easily selected from the PL map when the single hBN nanoparticles are arrayed; hence, it is an efficient method for finding the isolated spin defect in hBN nanoparticles by selecting the bright defects. As shown in Supplementary Fig. 12, there are mainly two categories of defects in our hBN nanoparticle sample (from the viewpoint of brightness), which exhibit very different brightness statistics. One category of nanoparticles exhibits very low brightness that is lower than 0.3 MHz, and the other one exhibits a much higher brightness that is higher than 0.8 MHz. Therefore, we chose the brightness threshold empirically as 0.5 MHz to select the bright spin defects in the same category.

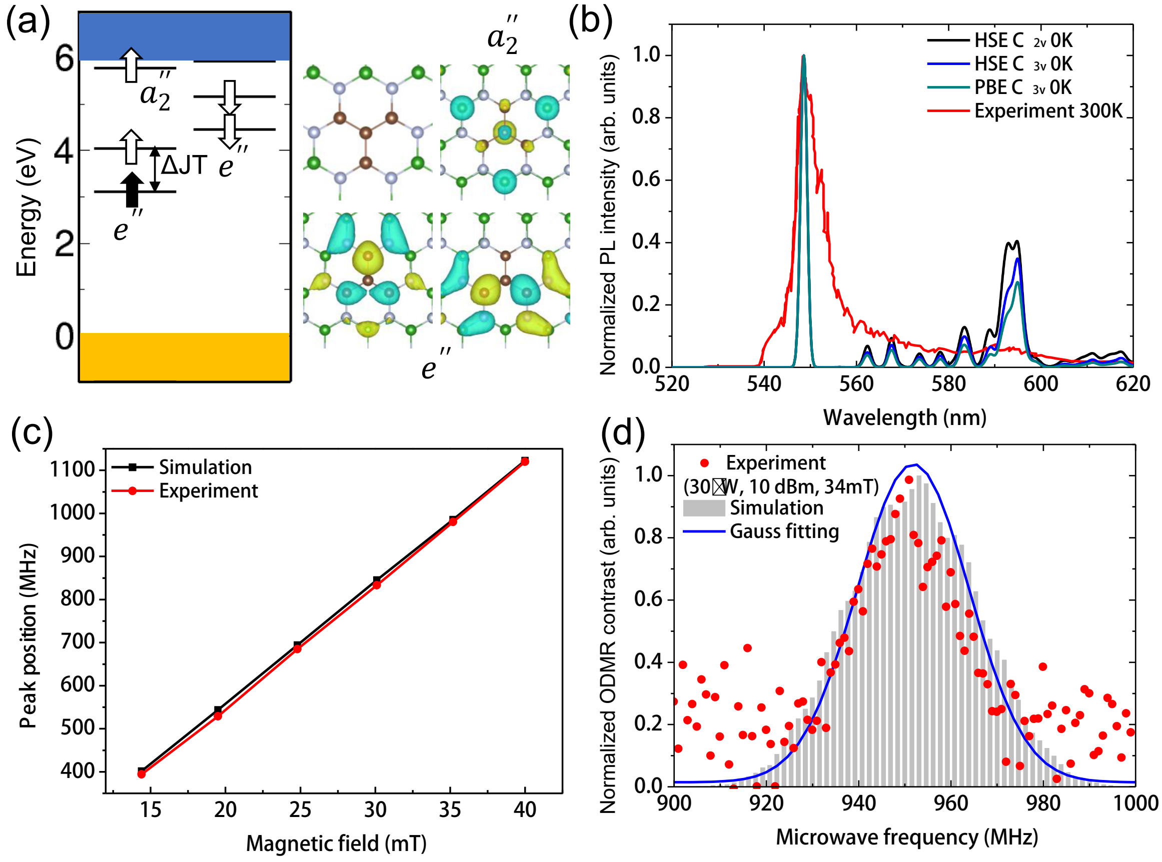

Supplementary Fig. 9: **Simulated electronic and optical properties of C_NC_{B3}.** (a) Kohn-Sham energy diagram of positive charged C_NC_{B3} with the localized wavefunction. The labeled atoms are the main contributors to hyperfine interactions. (b) Photoluminescence spectrum of C_NC_{B3} at 0K. (c) Dependence of ODMR resonance frequencies on the magnetic field. (d) Comparison between the simulated and experimental ODMR signals.

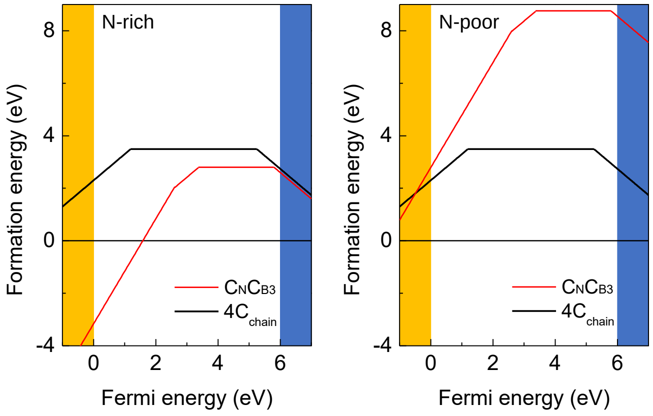

Supplementary Fig. 10: **Formation energy of C_NC_{B3}.** Formation energy as a function of the Fermi level in N-rich and N-poor conditions. The stable $4C_{chain}$ model here is for reference [9].There are 0, +1 and +2 stable charge states in the gap. Yellow and blue indicate the VB and CB of hBN.

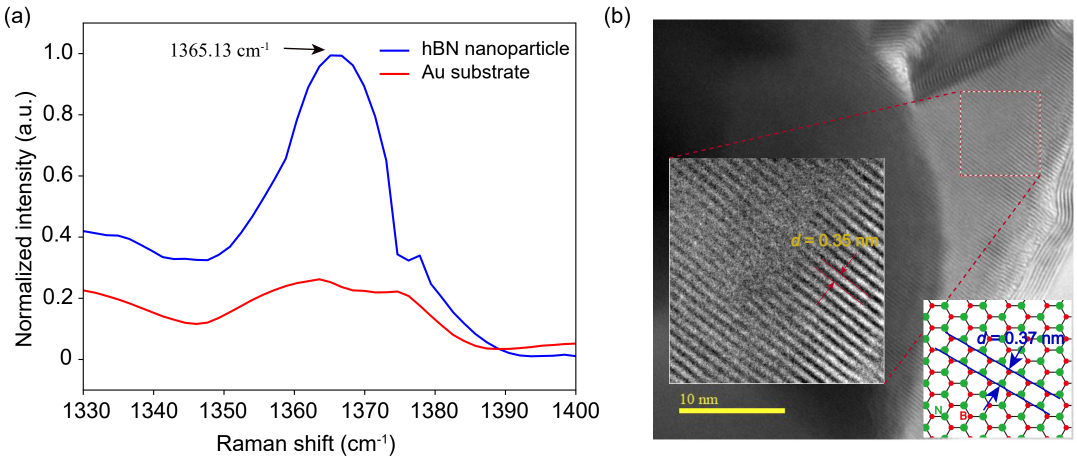

Supplementary Fig. 11: **Structural characterization of hBN nanoparticles.** (a) Raman spectrum of a nanoparticle containing a bright spin defect on a Au substrate. (b) Transmission electron microscope (TEM) image of the hBN nanoparticles. Inset: theoretical hBN lattice.

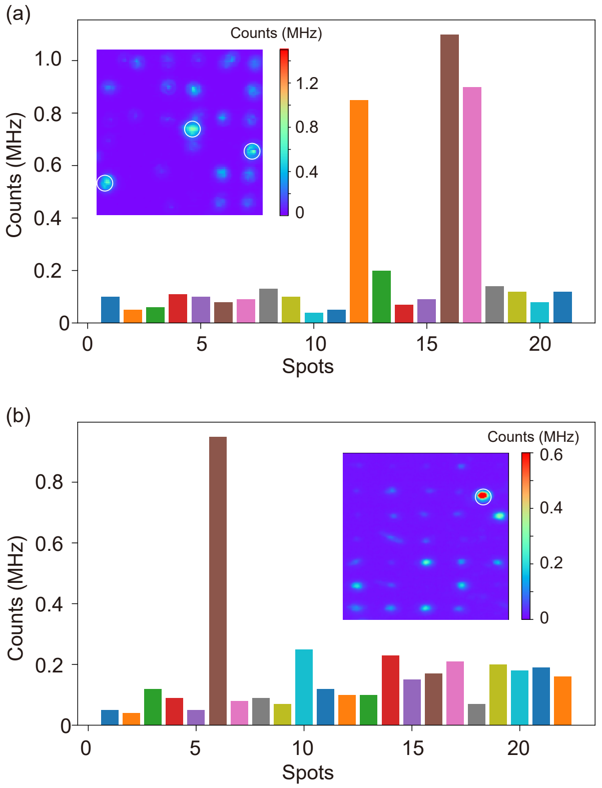

Supplementary Fig. 12: **Brightness statistics of the spots in the hBN defect arrays, where the spots circled in the inset are single spin defects.** The order of spots is detected from left to right and from top to bottom, except for some particularly dark spots. Inset: Confocal photoluminescence map of the hBN arrays with 100-μW laser excitation at 532 nm.

[1] Mendelson, N. *et al.* Identifying carbon as the source of visible single-photon emission from hexagonal boron nitride. *Nat. Mater.* **20**, 321-328 (2021).
[2] Stern, H. L. *et al.* Room-temperature optically detected magnetic resonance of single defects in hexagonal boron nitride. *Nat. Commun.* **13**, 618 (2022).
[3] Gottscholl, A. *et al.* Initialization and read-out of intrinsic spin defects in a van der Waals crystal at room temperature. *Nat. Mater.* **19**, 540-545 (2020).
[4] Chejanovsky, N. *et al.* Single-spin resonance in a van der Waals embedded paramagnetic defect. *Nat. Mater.* **20**, 1079-1084 (2021).
[5] Weston, L., Wickramaratne, D., Mackoit, M., Alkauskas, A. & Walle, C. Native point defects and impurities in hexagonal boron nitride. *Phys. Rev. B.* **97**, 214104 (2018)
[6] Smart, T., Li, K., Xu, J. & Ping, Y. Intersystem crossing and exciton-defect coupling of spin defects in hexagonal boron nitride. *Npj Comput. Mater.* **7**, 1-8 (2021)
[7] Auburger, P., & Gali, A. Towards ab initio identification of paramagnetic substitutional carbon defects in hexagonal boron nitride acting as quantum bits. *Phys. Rev. B* **104**, 075410 (2021).
[8] Li, K., Smart, T. & Ping, Y. Carbon trimer as a 2 eV single-photon emitter candidate in hexagonal boron nitride: A first-principles study. *Phys. Rev. Mater.* **6**, L042201 (2022)
[9] Li, S., Pershin, A., Thiering, G., Udvarhelyi, P. & Gali, A. Ultraviolet Quantum Emitters in Hexagonal Boron Nitride from Carbon Clusters. *J. Phys. Chem. Lett.* **13**, 3150-3157 (2022)
[10] Tran, T. T., Bray, K., Ford, M. J., Toth, M. & Aharonovich, I. Quantum emission from hexagonal boron nitride monolayers. *Nat. Nanotechnol.* **11**, 37-41 (2016).
